# A Lean Lady With Acanthosis Nigricans and Uncontrolled Diabetes Mellitus

**DOI:** 10.7759/cureus.11330

**Published:** 2020-11-04

**Authors:** Gururaja Rao, Yash V Chauhan, Premlata K Varthakavi, Nikhil Bhagwat

**Affiliations:** 1 Department of Endocrinology, Topiwala National Medical College and Bai Yamunabai Laxman Nair Charitable Hospital, Mumbai, IND

**Keywords:** type b insulin resistance, diabetes, acanthosis nigricans, steroids, insulin, antibodies, anti-insulin-receptor, systemic lupus erythematosis

## Abstract

A 47-year-old Asian Indian woman presented with uncontrolled hyperglycaemia and osmotic symptoms despite multiple oral antidiabetic medications and insulin. She had a history of recurrent oral ulcers, profound weight loss, and intermittent fever for one and a half years before the presentation. She had severe acanthosis nigricans, although her body mass index (BMI) was 14.6 kg/m^2^.

Her blood glucose remained uncontrolled despite very large dosages of intravenous insulin (more than 12,000 units daily). Evaluation for possible underlying collagen vascular diseases and malignancies were negative. Her serum insulin levels were high. She tested negative for anti-insulin antibodies but positive for anti-insulin-receptor antibodies. She improved with a pulse dose of intravenous methylprednisolone but relapsed within one month. A second pulse dose was given following which a complete remission of diabetes and regression of acanthosis was observed.

Type B insulin resistance, a rare cause of severe insulin resistance, may respond favourably to immunosuppressive therapy with high-dose methylprednisolone.

## Introduction

Type B insulin resistance is a rare cause of uncontrolled diabetes mellitus. It is caused by autoantibodies against insulin receptors and may often require very high doses of insulin. It is most commonly associated with systemic lupus erythematosus and other connective tissue disorders and less commonly with malignancies [1]. These associated diseases should be investigated and treated appropriately.

## Case presentation

A 47-year-old postmenopausal Asian Indian woman was referred for management of uncontrolled diabetes mellitus. She was diagnosed to have Type 2 diabetes mellitus three months prior to presentation when she was evaluated for osmotic symptoms. Her recent fasting and postprandial blood glucose levels were 305 and 416 mg/dL, respectively, despite high doses of multiple oral antidiabetic drugs (metformin 1,500 mg/day, glimepiride 6 mg/day, pioglitazone 15 mg/day). The hyperglycaemia had been unresponsive even to increasing dosages of insulin (multiple-dose injection insulin therapy with regular and neutral protamine Hagedorn (NPH) insulin of > 300 units/day). On inquiry, she had a history of recurrent oral ulcers, profound weight loss of 30 kgs over one and a half years, and intermittent fever without localising symptoms.

**Figure 1:**
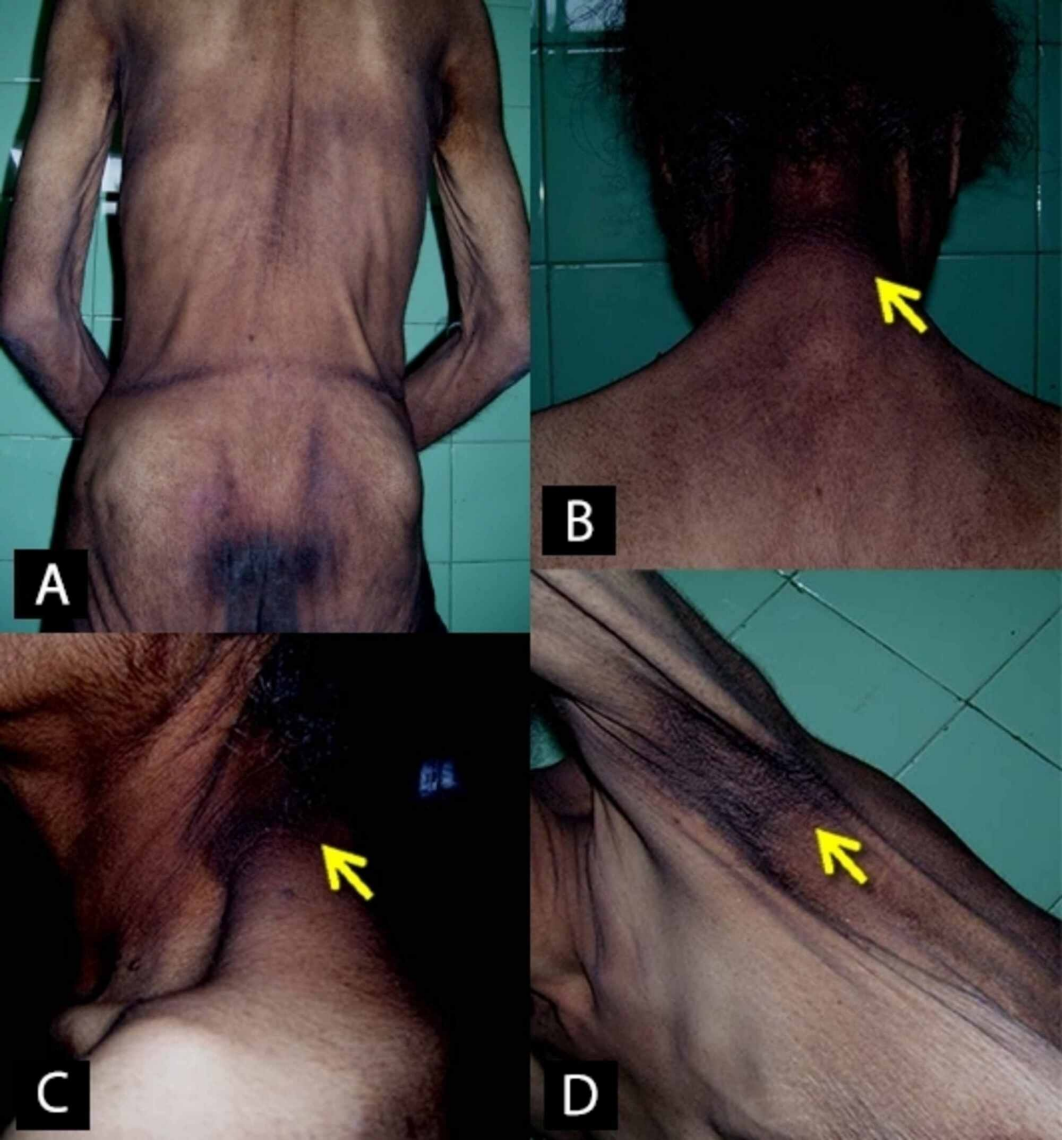
Extensive acanthosis nigricans over the body A: upper and lower back; B and C: neck, D: axillary region

On examination, she was emaciated. Her body mass index (BMI) was 14.6 kg/m^2^. She had extensive and severe generalised acanthosis nigricans over the nape and axillae with skin tags (Figure 1). Bilateral axillary lymph nodes were palpable. The systemic examination was otherwise unremarkable; in particular, there was no arthritis, serositis, or uveitis.

The patient was admitted and started on basal-bolus insulin therapy, along with metformin (1,500 mg/day) and pioglitazone (15 mg/day). Despite high doses of subcutaneous insulin, her blood glucose ranged between 400 - 600 mg/dL and her osmotic symptoms persisted. She was then started on intravenous insulin through an infusion pump. Despite increasing the insulin dose to more than 400 units/hour and 12,000 units daily, her blood glucose remained elevated.

On evaluation, she had low haemoglobin (8.8 gm%), leukopenia, normal platelet counts, and elevated erythrocyte sedimentation rate (40 mm/1st hr). Liver and renal function tests were normal. Hemoglobin A1c (HbA1c) at admission was 15.7%. Urinalysis revealed glycosuria but was negative for protein, ketone bodies, and blood. Fasting serum insulin done prior to initiating insulin was elevated (150 mU/L) and anti-insulin antibodies were negative (< 10% binding). Antinuclear antibody (ANA) was positive and showed a speckled appearance, but anti-double-stranded deoxyribonucleic acid (dsDNA) antibodies were negative. Anti-thyroid peroxidase antibodies were positive (1:100); however, she was euthyroid.

As our patient had severe acanthosis and uncontrolled diabetes despite very high dosages of insulin, syndromes of severe insulin resistance (defined as a daily insulin requirement of > 3 units/kg) were suspected [2]. Various causes of severe insulin resistance that were considered are listed in Table 1 [2-4].

**Table 1:** Classification of Syndromes of Severe Insulin Resistance HAIR-AN syndrome: hyperandrogenism (HA), insulin resistance (IR) and acanthosis nigricans (AN); HIV: human immunodeficiency virus; SHORT syndrome: short stature, hyperextensibility, hernia, ocular depression, Rieger anomaly, and teething delay

The various causes of severe insulin resistance that were considered: (Adapted from [2-4])
I. Primary insulin signaling defects
A) Generalised/insulin receptoropathies
- Type A insulin resistance syndrome
- Rabson-Mendenhall syndrome
- Leprechaunism/Donohue syndrome
- HAIR-AN syndrome
B) Partial – AKT2 mutation, SHORT syndrome (PI3KR1 mutation)
II. Secondary to adipose tissue abnormalities
A) Severe obesity
B) Lipodystrophy syndromes
1) Congenital
- Congenital generalised lipodystrophy (AGPAT2, BSCL2, CAV1, PTRF mutations)
- Familial partial lipodystrophy (LMNA, PPARG, ZMPSTE24, AKT2, CIDEC mutations)
2) Acquired
- Acquired generalised lipodystrophy (associated with other autoimmune diseases)
- Acquired partial lipodystrophy (HIV-associated, C3 nephritic factor-associated)
III. Immunologic, autoimmune
- Type B insulin resistance syndrome
- Antibodies to insulin
IV. Disorders of unknown aetiology – Pseudo-acromegalic insulin resistance

Given her age and evidence of associated autoimmunity, a clinical diagnosis of Type B insulin resistance syndrome (IRS) due to antibodies against the insulin receptor was considered (Table 2) [5-6]. Serial serum samples were preserved at -20°C for assaying anti-insulin-receptor antibodies later.

**Table 2:** Syndromes of Severe Insulin Resistance *Lipodystrophy includes congenital and acquired forms of complete and partial lipodystrophy IRS - insulin resistance syndrome

	Type A IRS	Type B IRS	Rabson-Mendenhall Syndrome / Donohue Syndrome	Lipodystrophy*	Our patient
Age at presentation	Adolescents or young adults	Most common in middle age; presenting age ranged from 10 to 68 years	First 1-2 years of life	Varies	47 years
Autoimmune features	-	+	-	-	+
Acanthosis nigricans	+	+	+	±	+
Dysmorphic features	-	-	+	-	-
Complete/partial loss of subcutaneous fat	-	-	-	+	-
Cause	INSR gene, Intracellular domain of tyrosine kinase	Autoantibodies against insulin receptors	Extracellular domain of tyrosine kinase	Genes affecting insulin signaling, caveolins, phospholipid biosynthesis, adipogenesis, and lipolysis	

Further investigations done to rule out connective tissue disorders and malignancies were unremarkable. X-ray of the chest, ultrasonography of the abdomen and pelvis, and skeletal survey for osteolytic lesions were normal. A pap smear was negative for atypical lesions. Biopsy of axillary lymph nodes showed reactive lymphadenopathy. Bone marrow aspiration and biopsy revealed micronormoblastic erythropoiesis. A whole-body ^18^F-fluorodeoxyglucose positron emission tomography (^18^FDG-PET-CT) scan did not show any abnormal pathology. Serum protein electrophoresis showed the absence of an M band. Antineutrophil cytoplasmic antibodies and anti-cardiolipin antibodies were negative, and serum complements (C3 and C4) were within the normal reference range.

She did not respond to oral prednisolone (1 mg/kg body weight daily) given for four weeks. In keeping with the clinical diagnosis of Type B IRS, she was pulsed with intravenous methylprednisolone (1 gm/day for three days), followed by a maintenance dose of oral prednisolone, 20 mg daily. After two weeks of post-pulse therapy, she achieved euglycemia without any anti-diabetic therapy.

Despite no carbohydrate restriction, she developed spontaneous episodes of hypoglycaemia, which resolved in two to three days. One month after pulse therapy, her blood glucose levels gradually started increasing, indicating a possible relapse. She was then given a single dose of 1 gm intravenous methylprednisolone followed by oral prednisolone and discharged at a dose of 15 mg daily.

**Figure 2:**
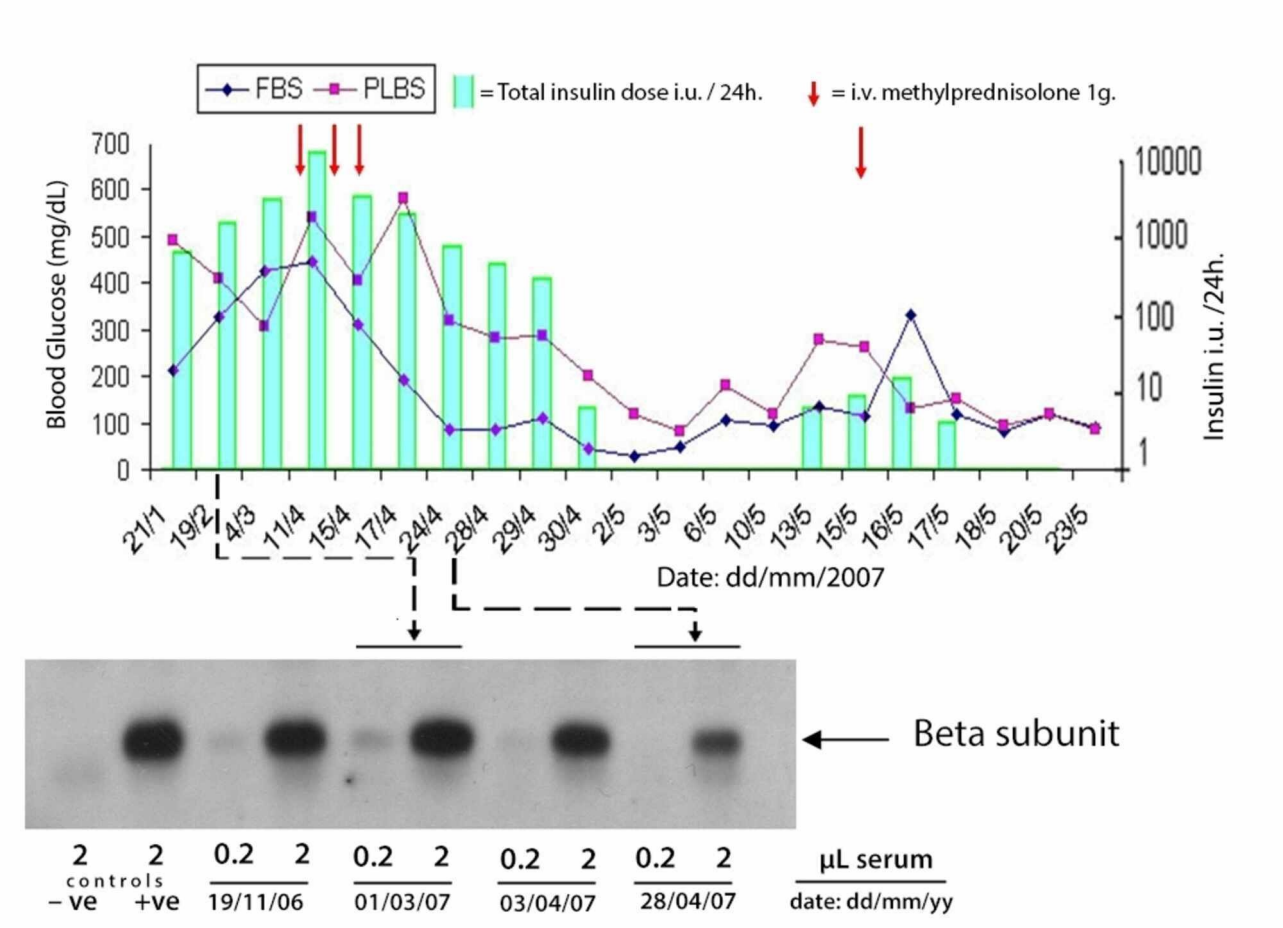
Insulin receptor antibodies (immunoprecipitation of soluble insulin receptors by patient’s serum) This figure demonstrates the resolution of hyperglycaemia correlating with a fall in the antibody titre. FBS: fasting blood sugar; PLBS: post-lunch blood sugar; - ve: negative control; + ve: positive control

The preserved serial serum samples were later sent to the Wellcome Trust-Medical Research Council (MRC) Institute of Metabolic Science, Cambridge, United Kingdom (UK) for assay of the anti-insulin-receptor antibodies using the immunoprecipitation technique. The antibodies were strongly positive, confirming the diagnosis of Type B IRS. The post-hoc analysis demonstrated a correlation between the fall in antibody titre and resolution of hyperglycaemia as shown in Figure 2.

**Figure 3:**
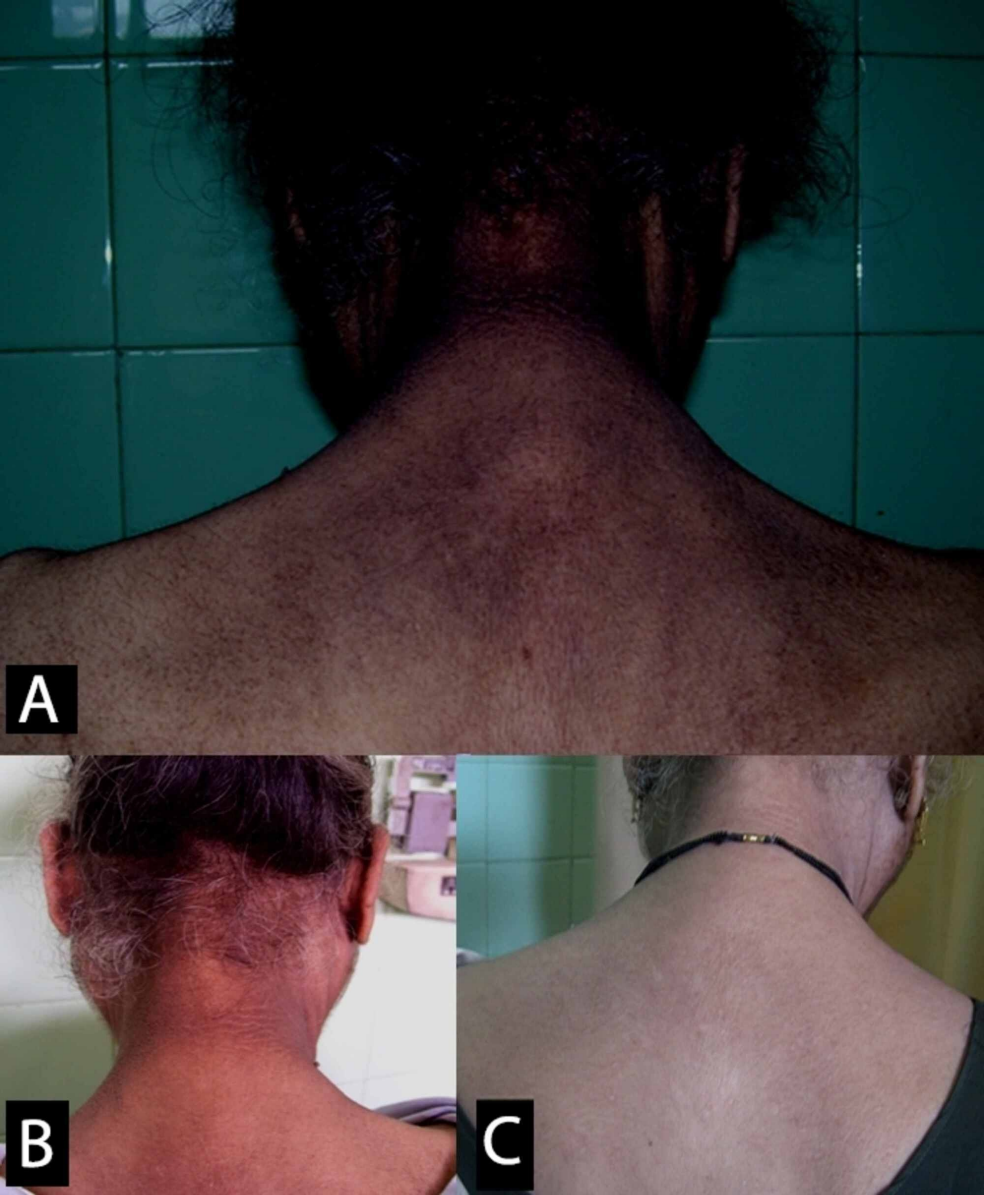
Resolution of acanthosis nigricans A: before treatment; B: at two weeks post-discharge; C: at the two-year follow-up

At the two-week follow-up, she was asymptomatic, had gained weight, and the acanthosis was regressing (Figure 3). Her blood glucose was within range without any anti-diabetic therapy. The oral steroids were gradually tapered following this visit.

Two years after the pulse therapy, she was still euglycemic, and the acanthosis had regressed completely. However, a year after her last follow-up, she was involved in a vehicular accident and passed away.

## Discussion

Type B IRS is an extremely rare cause of uncontrolled diabetes mellitus caused by autoantibodies to the insulin receptor. It is more common in middle-aged females, correlating with the incidence of other autoimmune diseases. The exact prevalence is not known [7-8]. By far, the most information comes from the cohort of 24 patients studied by the National Institutes of Health (NIH) over 28 years [9].

The concept of insulin receptor autoantibodies was first proposed by Flier and Kahn et al., who found a “factor” in the sera of six non-obese acanthotic women with reduced insulin responsiveness and then documented these antibodies a year later [10-11].

The antibodies are predominantly of IgG class and are believed to be inhibitory at high titres and stimulatory at low titres [1]. This may explain the spontaneous hypoglycaemia in our patient two weeks after the pulse therapy which resolved within two to three days.

The formation of antibodies is almost always part of another autoimmune disease, most often systemic lupus erythematosus (SLE). Our patient was positive for the antinuclear antibody (ANA) which was present in 83% of the NIH cohort [9]. However, our patient did not have any other features of SLE, had normal C3 and C4 levels, and was negative for anti-dsDNA, whilst 46% of the NIH study cohort fulfilled the SLE criteria. The NIH study findings are summarised in Table 3.

**Table 3:** Clinical Features of Type-B Insulin Resistance Syndrome ANA: antinuclear antibody; dsDNA: double-stranded deoxyribonucleic acid; ESR: erythrocyte sedimentation rate; IgA: immunoglobulin A; IgG: immunoglobulin G; NIH: National Institutes of Health; SLE: systemic lupus erythematosus Arioglu et al. [9]

Clinical Features of Autoimmunity	NIH Series	Our Patient
ANA	83%	+
Hypoalbuminemia	63%	+
Leukopenia	63%	+
Elevated ESR	75%	+
Proteinuria	54%	+
Serum IgG	46%	+
High serum anti-dsDNA	33%	-
Elevated serum IgA	25%	-
Alopecia	25%	-
Skin rash	21%	-
Low C3	21%	-
Nephritis	21%	-
Thrombocytopenia	21%	-
Enlarged salivary glands	17%	-
Arthritis	17%	-
Criteria of SLE	46%	-

Type B IRS may also be associated with other autoimmune connective tissue, thyroid, dermatologic, hematologic, and hepatic disorders, including primary biliary cirrhosis, scleroderma, dermatomyositis, overlap syndrome, and Hashimoto’s thyroiditis. It may be a paraneoplastic manifestation of certain malignancies, most commonly Hodgkin’s lymphoma and multiple myeloma [1, 9, 12-13].

Although no formal definition exists, it has been suggested that this syndrome may comprise of a triad of elevating fasting insulin, hyperadiponectinemia, and low or normal fasting triglyceride levels. These features in a patient with acanthosis nigricans, evidence of underlying autoimmune conditions, and uncontrolled hyperglycaemia may be considered diagnostic [1]. This hyperglycaemia often leads to a profound weight loss, and such patients are usually lean. Consistent with the hyperglycaemic NIH cohort, 80% of whom had a BMI < 30 kg/m^2^, our patient had a BMI of 14.6 kg/m^2^. Hypoglycaemia was the initial presentation in only 13% of the NIH cohort and 67% of them had a BMI > 30 kg/m^2^ [9]. Spontaneous hypoglycaemia has been reported in 23.5% of patients at some phase of their disease [14].

Management of hyperglycaemia in Type B IRS often involves large dosages of insulin. Management of hyperglycaemia in Type B IRS often involves large dosages of insulin; as much as 150,000 units/day has been reported in the past [15]. Our patient was still uncontrolled despite 12,000 units of insulin daily. Our patient also developed thrombophlebitis of the upper limb veins, possibly due to the phenolic preservative, which can be overcome by the administration of a more concentrated form of insulin. Insulin sensitizers, such as metformin and pioglitazone, may be used for glycaemic control; however, these patients often demonstrate persistent insulin resistance, despite high doses of these agents [16]. Liraglutide has also been successfully used to achieve complete glycaemic control [17].

Immunomodulation is the therapy of choice to induce remission (defined as the amelioration of hyperglycemia and discontinuation of insulin therapy) [18]. Various modalities have been employed in the past, albeit with extremely inconsistent remission and high mortality rates. These include high-dose corticosteroids, plasmapheresis, intravenous immunoglobulins, and other immunosuppressants, such as mycophenolate mofetil, cyclophosphamide, cyclosporine, and azathioprine [1, 9, 19-20]. More recently, a standardised protocol has been developed by the NIH consisting of high-dose corticosteroids, cyclophosphamide, and rituximab as induction agents, followed by maintenance therapy with azathioprine or cyclosporine. Significantly higher remission rates (86.4% vs 41.4%), lower duration to remission (five months vs 30 months), and lower mortality (0% vs 44.8%) have been reported with this protocol compared to the historical NIH cohort when adjusted for a similar time period [7, 18]. Our patient was fortunate enough that, in an extremely resource-limited setting, she responded favourably to high-dose corticosteroids and remained in remission for at least two years without the need for more expensive treatment, such as rituximab or cyclophosphamide.

At the time of publication of the NIH study, three of the nine patients that were still being followed up were positive for the antibody. In contrast, in our patient, consistent with other literature, improvement in dysglycaemia correlated with a fall in titre of antibodies [9, 13]. Without treatment, a spontaneous resolution was noted in 33% of the NIH cohort, while 25% had spontaneous hypoglycaemia. However, the overall cohort had a striking 54% mortality, and of note, 23% of those died from complications of hypoglycaemia.

## Conclusions

Type B insulin resistance has a predilection for middle-aged females. These patients typically present with extensive acanthosis despite a lean phenotype. Possible underlying connective tissue diseases and malignancies should be ruled out in these patients. It can also present as spontaneous hypoglycaemia and is one of the two recognised causes of autoimmune forms of hypoglycaemia, the other being insulin autoimmune syndrome. Immunosuppressive therapy is the modality of choice to induce remission and includes a multitude of therapies, including high-dose corticosteroids, as seen in our case. More recently, elaborate rituximab and cyclophosphamide-based regimens have recently been developed and shown to have better remission rates.
